# Inference of alveolar capillary network connectivity from blood flow dynamics

**DOI:** 10.1152/ajplung.00025.2024

**Published:** 2024-09-25

**Authors:** Kerstin Schmid, Andy L. Olivares, Oscar Camara, Wolfgang M. Kuebler, Matthias Ochs, Andreas C. Hocke, Sabine C. Fischer

**Affiliations:** ^1^Fakultät für Biologie, Center for Computational and Theoretical Biology, Julius-Maximilians-Universität Würzburg, Würzburg, Germany; ^2^Sensing in Physiology and Biomedicine (PhySense), Department of Information and Communication Technologies, Universitat Pompeu Fabra, Barcelona, Spain; ^3^Institute of Physiology, Charité—Universitätsmedizin Berlin, Berlin, Germany; ^4^Institute of Functional Anatomy, Charité—Universitätsmedizin Berlin, Berlin, Germany; ^5^Department of Infectious Diseases, Respiratory Medicine and Critical Care, Charité - Universitätsmedizin Berlin, Corporate Member of Freie Universität Berlin and Humboldt-Universität zu Berlin, Berlin, Germany; ^6^German Center for Lung Research (DZL), Berlin, Germany

**Keywords:** arteriole connectivity, computational fluid dynamics, data-driven in silico modeling, functional morphology, pulmonary microvasculature

## Abstract

The intricate lung structure is crucial for gas exchange within the alveolar region. Despite extensive research, questions remain about the connection between capillaries and the vascular tree. We propose a computational approach combining three-dimensional (3-D) morphological modeling with computational fluid dynamics simulations to explore alveolar capillary network connectivity based on blood flow dynamics. We developed three-dimensional sheet-flow models to accurately represent alveolar capillary morphology and conducted simulations to predict flow velocities and pressure distributions. Our approach leverages functional features to identify plausible system architectures. Given capillary flow velocities and arteriole-to-venule pressure drops, we deduced arteriole connectivity details. Preliminary analyses for nonhuman species indicate a single alveolus connects to at least two 20-µm arterioles or one 30-µm arteriole. Hence, our approach narrows down potential connectivity scenarios, but a unique solution may not always be expected. Integrating our blood flow model results into our previously published gas exchange application, Alvin, we linked these scenarios to gas exchange efficiency. We found that increased blood flow velocity correlates with higher gas exchange efficiency. Our study provides insights into pulmonary microvasculature structure by evaluating blood flow dynamics, offering a new strategy to explore the morphology-physiology relationship that is applicable to other tissues and organs. Future availability of experimental data will be crucial in validating and refining our computational models and hypotheses.

**NEW & NOTEWORTHY** The alveolus is pivotal for gas exchange. Its complex, dynamic nature makes structural experimental studies challenging. Computational modeling offers an alternative. We developed a data-based three-dimensional (3-D) model of the alveolar capillary network and performed blood flow simulations within it. Choosing a novel perspective, we inferred structure from function. We systematically varied the properties of vessels connected to our capillary network and analyzed simulation results for blood flow and gas exchange to obtain plausible vessel configurations.

## Graphical abstract

**Figure:**
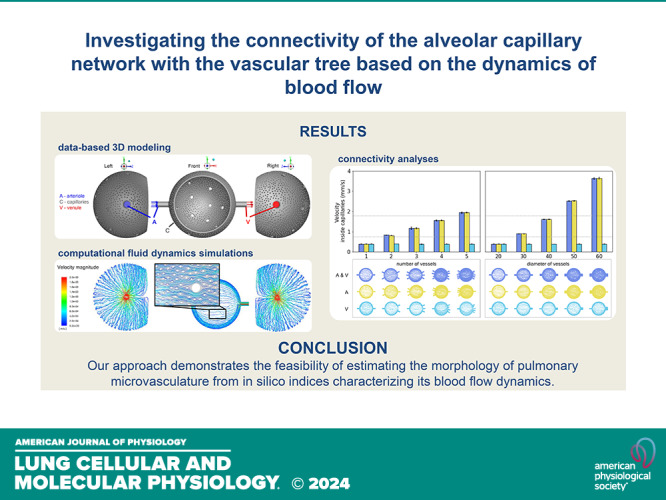

## INTRODUCTION

Morphology and physiology are closely intertwined for organ function. In the lung, the minimal functional unit, the alveolus, is structured and composed for the vital exchange of carbon dioxide and oxygen between the air space and capillary blood system. This demand requires maximizing surface area while minimizing barrier thickness. In addition, efficient blood flow is essential, necessitating minimal resistance to flow but allowing sufficient contact time for effective gas exchange at the same time.

Despite the inherent relationship between structure and function, experimental studies often consider them separately. Traditional morphological studies (1–3) rely on invasive techniques for organ fixation, often causing tissue distortion and compromising accuracy (4–9). Physiology research on the whole lung scale can use noninvasive methods (e.g., see Refs. 10 and 11). Yet, investigating processes of the alveolar compartment, such as blood flow distribution, remains a major challenge.

Computational modeling offers a unique avenue to integrate morphological and physiological research. In pulmonary research, computational fluid dynamics simulations have been used to study the air flow in the conducting airways (12). Typically, a combination of computer tomography (CT) imaging data and in silico modeling has enabled building accurate anatomical representations of the respiratory tree to predict organ function (reviewed in Ref. 13). With respect to the pulmonary microcirculation, computational approaches have included increasingly sophisticated two-dimensional (2-D) and three-dimensional (3-D) models of capillary networks to predict the distribution of blood flow (14–16). These approaches have primarily focused on the impact of morphological details on physiological output.

In this work, we demonstrate how physiological parameters can provide morphological insight. Our approach combines 3-D morphological modeling of an alveolus and the alveolar capillary network (ACN) with blood flow simulations using computational fluid dynamics (CFD). Our focus is on uncharted morphological aspects of the ACN’s connection to the vascular tree. We link the 3-D ACN model to generic arteriole and venule models, varying their numbers and diameters. We demonstrate how the choice of boundary conditions and model parameters such as the fluid viscosity influence the CFD simulation results, including capillary flow velocity and arteriole-to-venule pressure drop. With simulation settings based on findings from disparate experimental studies, our model produces results that are within the range of literature reference values. We illustrate how the physiological parameters—capillary flow velocity and pressure drop—can be used to infer structural details about the linkage between the ACN and arterioles or venules. Integration of the blood flow dynamics into our interactive model for gas exchange (17) provides a link of our results to gas exchange efficiency. It is crucial to emphasize that the success of our approach relies on the availability of appropriate experimental data for validation.

## MATERIALS AND METHODS

**Figure 1.**
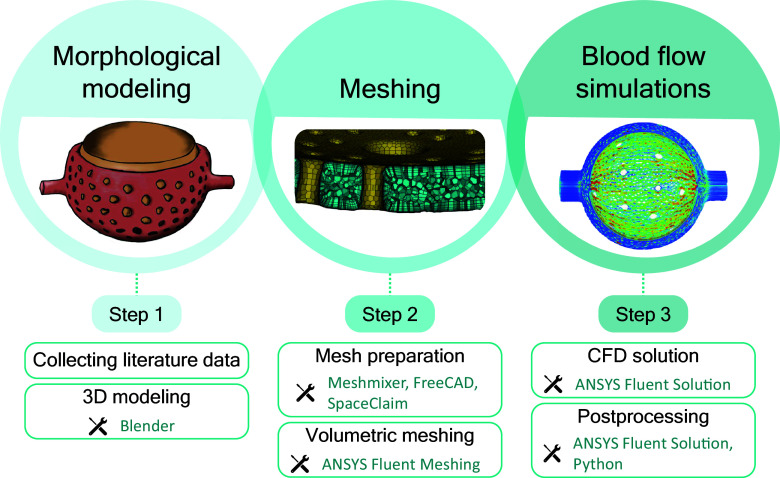
The methodological concept of this work combines morphological in silico modeling of the alveolus and the alveolar capillary network with blood flow simulations in three main steps. First, relevant morphological data were collected from existing literature. Using the software Blender, three-dimensional (3-D) models of the alveolus and the alveolar capillary network were generated. Next, these models underwent mesh preparation using Meshmixer, FreeCAD, and SpaceClaim to ensure compatibility with the Ansys software. Ansys Fluent Meshing was used to create volumetric meshes for computational fluid dynamics simulations. Finally, the simulations were conducted with Ansys Fluent Solution. Postprocessing, including data extraction and analysis, was performed using Ansys Fluent Solution and Python scripting.

For this integrative study, a number of specialized software tools were used in a multistep workflow (Fig. 1). We created 3-D models of the ACN based on morphometric data from the literature. These models served as templates for building high-quality finite-element meshes, which were subsequently used to run CFD simulations to predict blood flow dynamics.

### Morphological Modeling

The true-to-scale, three-dimensional models of the capillary network around a single alveolus were manually created with the modeling software Blender v.3.3 (18) (Supplemental Fig. S.1). Emphasis was placed on ensuring that the scale and size ratios correspond to quantitative morphological data found in the literature. These measurements are taken from fixed lungs (Supplemental Table S.1).

#### Alveolar base.

**Figure 2.**
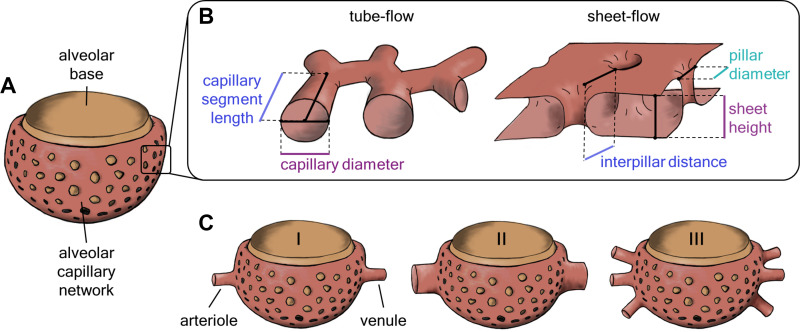
*A*: the alveolar capillary network is built around an open 3/4 spheroid, the alveolar base. *B*: two different concepts of the capillary bed geometry, the tube-flow and the sheet-flow, were compared. A tube-flow capillary network is parameterized by the length and diameter of the capillary segments. In contrast, the sheet-flow capillary bed is defined by the height of the sheet, the diameter of the tissue pillars, and their distance from each other. *C*: the capillary network was connected to different configurations of arterioles and venules. Starting from the default configuration (I) of one arteriole and one venule, both 20 µm in diameter, either the diameter (II) or the number (III) of vessels was varied. [Figure reproduced from a thesis dissertation under CC BY-NC license (69).]

Initially, a basic geometry of an alveolus was created, which will be referred to as “alveolar base” in the following. It was constructed as an open 3/4 spheroid with an opening, the so-called alveolar mouth (Fig. 2*A*). The diameter or volume of the spheroid, and its depth and the diameter of the opening were based on published stereological measurements, making them the input parameters for our modeling process. On the other hand, we used a separate set of parameters for evaluation, comparing them with reference values from the literature. These “output parameters” encompass the surface area and volume or diameter of the alveolar base. All parameter values are listed in Table 1 in results.

**Table 1.** Comparison of in- and output parameter values of 3-D geometrical models Alveolus_D_ and Alveolus_V_ with morphometric measurements from the literature

Parameter	Literature	Alveolus_D_ Model	Alveolus_V_ Model
Modeling (input)			
Alveolus—Shape	3/4 spheroid, truncated cone, 1/4 spheroid, …(19)	3/4 spheroid	3/4 spheroid
Alveolus—Diameter, µm	225 (20), 232 (19)	225	–
Alveolus—Volume, 10^6^ µm^3^	4.6 (19), 4.2 (21), 3.4 (20)	–	4.6
Pores of Kohn—Number	17 (22)	17	17
Pores of Kohn—Diameter, µm	7–19 (22), 7.2–16.5 (23)	12.3	12.3
Alveolar Mouth—Diameter, µm	181 (19), 220 (24)	181	181
Evaluation (output)			
Alveolus—Diameter, µm	225 (20), 232 (19)	–	216
Alveolus—Volume, 10^6^ µm^3^	4.6 (19), 4.2 (21), 3.4 (20)	5.3	–
Alveolus—Depth, µm	174 (19)	178	167
Alveolus—Surface Area, 10^5^ µm^2^	1.2 (19, 20), 2.1 (25)	1.52	1.39
Alveolar Mouth—Area, 10^5^ µm^2^	0.3 (19)	0.26	0.24

The difference between the two models is that the input parameters for Alveolus_D_ included the diameter and for Alveolus_V_ the volume of the alveolus. By comparing the resulting output parameters with measurements from the literature, the models were evaluated. References: 19–25.

#### Alveolar capillary network.

The capillary network was built around this alveolar base. We considered two alternative concepts of the morphology of the alveolar capillary bed that have been proposed in the literature, the tube-flow (3) and the sheet-flow (26) (Fig. 2*B*). In the tube-flow model, the capillary bed is considered as a network of cylindrical tubes with a defined diameter and length. In the sheet-flow model, the capillary bed is regarded as a continuous sheet formed by two endothelial layers. These layers are held apart by connective tissue and cellular pillars at a specific height, i.e., the thickness of the sheet. The diameter and spacing of the pillars further determine the structure of the capillary bed.

We followed different modeling approaches for the tube-flow and the sheet-flow models of the ACN, based on correspondingly different input parameters. For the tube-flow model, the diameter and length of capillary segments were aligned with measurements from Mühlfeld et al. (3). The sheet-flow model was built taking into account the height of the sheet, the diameter of the tissue pillars, and their distance from each other. Existing literature only provides measurements of cats for these parameters (27). To create a sheet-flow model that matches the morphological proportions of the human ACN, the following assumptions were made: the height of the capillary sheet corresponds to the diameter of capillary tubes and the distance between pillars corresponds to the capillary segment length. The pillar diameter was derived by assuming the same ratio between pillar diameter and interpillar distance as observed in cats, roughly 0.5 (27). All parameter values are listed in Table 2 in results. Further details on the modeling workflow in Blender are provided in Supplemental Section S.1. For evaluation purposes, the volume and surface area of the 3-D ACN models were compared with reference values from morphological studies.

**Table 2.** In silico models of the human ACN have lower surface area and volume compared to corresponding estimates from morphometric studies

Parameter	Literature	Alveolus_D_ Model		Alveolus_V_ Model	
		Sheet-Flow	Tube-Flow	Sheet-Flow	Tube-Flow
Modeling (input)					
Capillary diameter/sheet height, µm	6.3 (3)	6.34	6.31	6.36	6.3
Capillary segment length/interpillar distance, µm	5.9 (3)	6.17	5.75	6.33	5.54
Capillary pillar diameter, µm	3*	3.1	–	3.23	–
Evaluation (output)					
Capillary volume, 10^5^ µm^3^	8.2** (3), 8.8** (1)	7.12	6.09	6.32	5.54
Capillary surface area, 10^5^ µm^2^	5.4** (3), 5.3** (1), 3.0** (25)	3.42	3.23	3.12	2.93
Capillary surface area, available for gas exchange, 10^5^ µm^2^		1.11	1.09	0.98	0.95

Tube-flow and sheet-flow models of the alveolar capillary network (ACN) around the Alveolus_D_ and Alveolus_V_ models, respectively, were built based on literature reference values for the input parameters. Output parameters were measured after the modeling process and compared with literature values for evaluation purposes. References: 1, 3, 25.

* Derived from interpillar distance assuming a ratio between pillar diameter and interpillar distance of 0.5; **Measurements for the entire lung were converted to an alveolus, assuming 480 million alveoli in the human lung (21).

#### Connectivity models.

We set out to investigate the linkage of the ACN to the rest of the pulmonary vascular tree. There is no distinct perfusion unit as the ACN is a continuum connected to multiple arterioles and venules and spanning multiple alveoli (28). To characterize the tissue structure, it is therefore useful to determine the ratio of the number of arterioles and venules to alveoli. Specifications for the diameter of arterioles and venules in the literature range from 13 to 30 µm (29–32). In addition, it was also observed how alveolar capillaries branch off from rather large arteries with diameters of up to 100 µm (29). Based on this information, we created different configurations of arterioles and venules connected to our ACN model for the connectivity analyses (Fig. 2*C*). The default configuration was one arteriole and one venule, both with a diameter of 20 µm and positioned opposite each other. Starting from this, we created two different sets of models. In the first set, we varied the diameter of the vessels connected to the ACN from 20 µm to 60 µm in increments of 10 µm. This was done either symmetrically or only for one of the vessel types (arteriole or venule) at a time. In the second set, the number of connected arterioles and venules was increased from one to five. Again, the changes were made both symmetrically and for each vessel type individually.

### Volumetric Mesh Preparation

**Figure 3.**
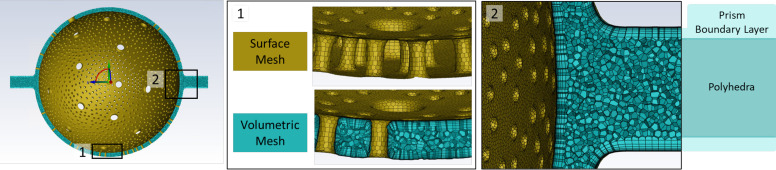
A finite volume mesh generated with the Ansys Fluent Meshing software. The surface mesh (gold) defines the capillary wall and inlet and outlet surfaces as they are given by the Blender model. The volumetric mesh (turquoise) consists of a boundary layer of prism elements near the wall and polyhedral elements inside the volume. The prism elements of the boundary layer increase in thickness from the wall toward the polyhedral elements. [Figure reproduced from a thesis dissertation under CC BY-NC license (69).]

The models of the ACN created in Blender were hollow bodies with their surface described by an average of 2.1 × 10^6^ nodes and faces and 4.2 × 10^6^ edges. For blood flow simulations using CFD, the fluid domain, i.e., the volume inside the capillary, must be defined by a discrete volumetric mesh. Hence, a finite volume (FV) mesh combines both components: a surface mesh, describing the wall of the geometry and inlet and outlet faces and the volumetric mesh defining the interior of the geometry (Fig. 3). To be able to simulate the interaction of the fluid with the wall, a particularly high resolution of the mesh elements close to the wall is necessary. The volumetric mesh is therefore divided into a high-resolution boundary layer of prism elements near the wall and a coarser layer of polyhedral elements in the center.

To serve as a template for FV meshes, the Blender geometries of the ACN first had to be processed in several steps using different software programs. First, the geometries were exported from Blender as stereolithography (STL) files. In STL files, the surface of a 3-D object is defined by a mesh of triangular faces. With Autodesk Meshmixerversion 3.5.474 (33), the resolution of this surface mesh was then reduced to roughly 500,000 triangles without distorting the geometry. In a next step, the STL files were converted to a computer-aided design (CAD) file using the open-source software FreeCAD version 0.20.1 (34). In this format, they were compatible with Ansys’ modeling software SpaceClaim, where the ACN geometry was organized into inlet, wall, and outlet zones. Finally, the FV mesh was created with the Ansys Fluent Meshing software using the guided workflow “watertight geometry.” This is the primary workflow to generate FV meshes from clean boundary meshes, i.e., geometries without any leakage, as was the case with our geometries. More details on the meshing workflow are included in Supplemental Section S.2 including Supplemental Table S.2. The quality of the meshes was quantified in terms of the parameters inverse orthogonal quality, size change, and aspect ratio (Supplemental Section S.3 including Supplemental Table S.3).

### Computational Fluid Dynamics

We simulated blood flow in the morphometric models described in *Connectivity models* using computational fluid dynamics (CFD). The CFD study was performed with the software Ansys Academic Research CFD, release 2022 R2 (35) (Supplemental Section S.4). A physical model describing the fluid flow was solved numerically in the domain of the ACN models (see *Connectivity models*). We evaluated and estimated the quality of our FV meshes, and our CFD solution (Supplemental Section S.5). For the connectivity analysis, we performed the simulations on four replicates of the ACN. We generated two meshes from scratch and additionally used the mirrored versions of the two.

#### CFD solution.

The three-dimensional flow of a fluid was described by a system of second-order nonlinear partial differential equations based on the conservation of mass (1) and momentum (2, 36). The equation for the conservation of mass (or continuity equation) is expressed as follows:

(*1*) $$\frac{\partial \rho }{\partial t}+\nabla \cdot (\rho \vec{v})=0,$$

where ∇ is the divergence operator applied to both the density ρ and the velocity vector $\vec{v}$. In the case of an incompressible fluid, the rate of density change $\frac{\partial \text{ρ}}{\partial t}$ can be neglected. The conservation of momentum was expressed as follows:

(*2*) $$\frac{\partial (\text{ρ}\vec{v})}{\partial t}+\nabla (\text{ρ}{\vec{v}}^{2})=-\nabla p+\nabla (\bar{\bar{\tau }})+\text{ρ}\vec{g},$$

where *p* is the static pressure, $\bar{\bar{\tau }}$ is the stress tensor that depends on molecular viscosity, and $\text{ρ}\vec{g}$ is the gravitational body force.

In Ansys Fluent Solution, these equations were represented as algebraic expressions in discrete domains of space (finite-volume method) and a pressure-based solver was used to solve them iteratively for a steady state. An estimation of the Reynolds number for the flow in our model resulted in 0.0076. Therefore, the viscous model was set to laminar flow. Vessel walls were assumed rigid, and a no slip boundary condition was applied at the walls. A detailed list of simulation settings made in Ansys is given in Supplemental Section S.4. The target values recorded were the mass-weighted average of flow velocity within capillaries and the pressure drop, calculated from the mass-weighted averages of static pressure at the inlet and outlet surfaces. In addition, the global mass balance was monitored for every iteration during the simulation. Convergence criteria were met when the scaled and normalized residual values fell below the threshold of 1 × 10^−5^. Furthermore, it was manually controlled that all target values had reached a constant value and that the global mass balance (defined as the sum of mass flow rates of all inlet and outlet faces) had leveled out around 0. More information on the convergence criteria are included in Supplemental Section S.5 including Supplemental Fig. S.2.

#### Fluid properties and boundary conditions.

Blood was approximated using two approaches: First, as a Newtonian fluid with a constant viscosity of 2 cP. Second, as a non-Newtonian fluid using the Carreau viscosity model. This model describes the apparent viscosity η of a fluid as a function of shear rate γ as follows:

(*3*) $$\eta =\eta_{\infty }+({\text{η}}_{0}-{\text{η}}_{\infty }){\left[1+{\text{γ}}^{2}{\text{λ}}^{2}\right]}^{(n-1)/2},$$

where η_0_ = 0.056 Pa·s (56 cP) and η_∞_ = 0.0035 Pa·s (3.5 cP) are the viscosity values at zero and infinitely high shear rates, λ = 1.902 s is a time constant, and *n* = 0.3568 is the power-law index. The parameter values appropriate for blood conditions were adopted from Albors et al. (37).

Fluid density was set constant at 1,050 kg/m^3^. Boundary conditions were set at 0.0023 m/s inlet velocity [2.3 mm/s (38)] and 1,127 Pa outlet pressure [11.5 cmH_2_O (39)].

#### Postprocessing.

In Ansys, the results were visualized in the 3-D model using pathlines or heatmaps. A pathline represents the path of a particle over time. They were color-coded according to the velocity magnitude. Heatmaps illustrated the distribution of static pressure in the model. Analysis of the quantitative simulation output and the presentation of the results by graphs was done in Python v.3.9.12. Means and standard deviations of four technical replicates are given. Regression analyses were performed on the relationship between target values and arteriolar volume. To facilitate an easier comparison of our simulation results with literature values, we converted the units used in Ansys, which were m/s for velocity and Pa for pressure, to mm/s and mmHg, respectively.

#### Sensitivity analyses.

Using the Newtonian fluid model, the boundary conditions inlet velocity and outlet pressure, and the fluid properties density and viscosity were varied separately while maintaining the standard settings used in our main experiments (Supplemental Table S.4). Regarding the boundary conditions and fluid density, we selected value ranges of reasonable magnitude, with our standard values positioned approximately in the middle of these ranges. Specifically, we varied the inlet velocity from 0.5 mm/s to 3 mm/s in 0.25 mm/s increments, the outlet pressure from 500 Pa (3.75 mmHg) to 1,500 Pa (11.25 mmHg) in 100 Pa increments and density between 500 kg/m^3^ and 1,500 kg/m^3^ in 100 kg/m^3^ increments. The viscosity value range was chosen to encompass both the viscosity value of our Newtonian fluid model (2 cP) and all values applicable within the non-Newtonian viscosity model. It ranged from 1 cP to 56 cP in steps of 5.5 cP.

## RESULTS

### Evaluation of 3-D Morphological Models of Alveolar Capillary Network

The first milestone of our work was a 3-D model of the ACN around an alveolus based on published morphological measurements from fixed lungs. When modeling the alveolar base, the following behavior was observed: if the model was based on the literature values for the diameters of the alveolus and the alveolar opening, the alveolar volume exceeded the respective measurement from the same study (19) (Supplemental Fig. S.3). Conversely, an alveolus model with the appropriate volume had a smaller diameter than specified in the literature (Table 1). For the time being, we continued with two different models, either based on the diameter (Alveolus_D_) or on the volume (Alveolus_V_).

Next, we added the ACN to the in silico alveolus model. We compared tube-flow and sheet-flow models of capillary beds around the Alveolus_D_ and Alveolus_V_ base models (Table 2). The volume and surface area of these models were found to be lower than the values reported in the literature. In general, however, the Alveolus_D_ ACN models were closer to the literature values than the Alveolus_V_ ACN models and the same tendency applied to the sheet-flow models compared with the tube-flow models. Since the Alveolus_D_ sheet-flow model is also easier to implement, we considered it the best candidate for further work.

### Blood Flow Simulations in a 3-D Model of the Alveolar Capillary Network

**Figure 4.**
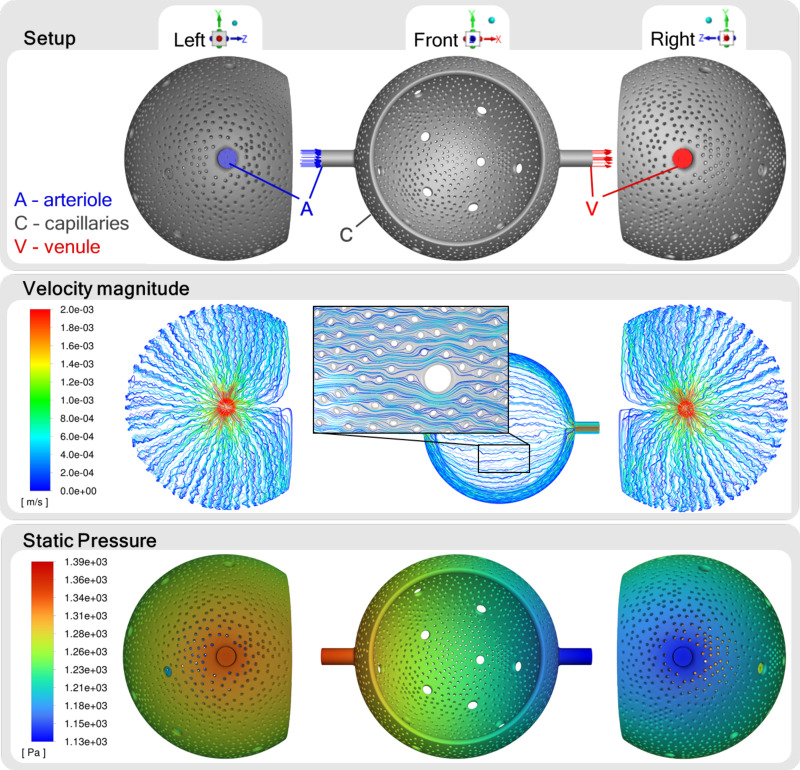
Results of computational fluid dynamics simulations in the three-dimensional (3-D) alveolar capillary network model based on the Alveolus_D_ model. The capillary network was connected to one arteriole (inlet) and one venule (outlet), each with a diameter of 20 µm. Blood was approximated as a Newtonian fluid. Pathlines are color-coded according to the velocity magnitude, obtaining a mean flow velocity within the capillaries of 0.39 mm/s. The pressure decreased continuously from its maximum at 1,345 Pa (10.09 mmHg) to its minimum at 1,127 Pa (8.45 mmHg). [Figure reproduced from a thesis dissertation under CC BY-NC license (69).]

Next, we used CFD simulations to predict blood flow in our 3-D sheet-flow model of the ACN (Fig. 4). The simulation results showed that flow velocity inside the capillaries was lower compared with inside the inflow (arteriole) and outflow (venule) vessels for the standard configuration with a diameter of 20 µm each (Supplemental Fig. S.4). In addition, the flow directions parallel to the orientation of these vessels were strongly preferred. This arrangement of arteriole, ACN, and venule resulted in some areas of the capillary network being more perfused than others. Regarding the mean flow velocity within the capillary network, simulations with Newtonian and non-Newtonian viscosity models yielded consistent results of 0.39 mm/s ± 0.004 and 0.4 mm/s ± 0.003, respectively. In both cases, the pressure was evenly distributed across the model from the maximum at the inlet surface to the minimum at the outlet surface. However, the pressure drop in the Newtonian simulations, with a value of 1.64 mmHg ± 0.043, was markedly lower than the 3.54 mmHg ± 0.08 observed in the non-Newtonian simulations.

**Figure 5.**
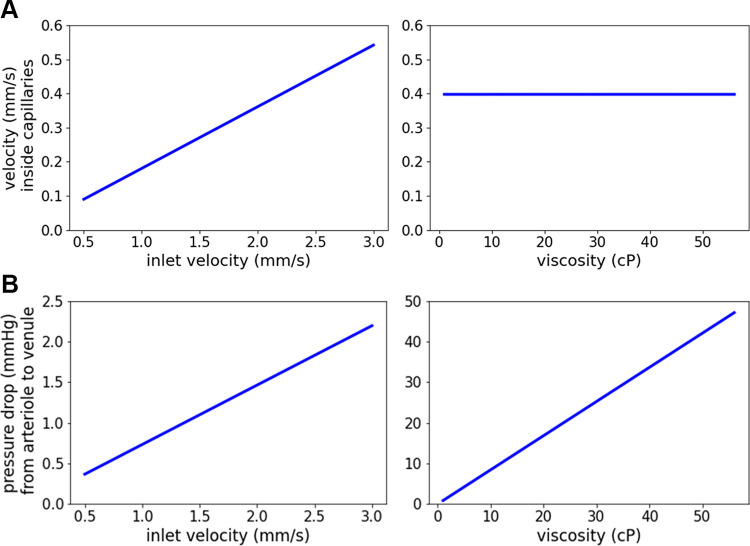
Sensitivity analysis for the effects of inlet velocity and fluid viscosity on the target values: mean flow velocity within capillaries (*A*) and pressure drop from arteriole to venule (*B*). Simulations were performed in the default model with one arteriole and one venule, both 20 µm in diameter. The inlet velocity was varied from 0.5 mm/s to 3 mm/s in steps of 0.25 mm/s. The viscosity was varied between 1 cP and 56 cP in steps of 5.5 cP. The Alveolus_D_ model was used. We show the mean values at the center position of the alveolar capillary network (ACN). [Figure reproduced from a thesis dissertation under CC BY-NC license (69).]

To evaluate the effect of variations in boundary conditions at the inlet and outlet, and in the fluid properties of density and viscosity on the simulation results, we performed sensitivity analyses. The flow velocity at any point in the model depends on the mass flow rate and the fluid density (*Eq. 1*). The mass flow rate is determined by the inlet velocity boundary condition. Accordingly, a linear relationship could be observed between the inlet velocity and the mean velocity within capillaries (Fig. 5*A*). The static pressure at the outlet (data not shown) and the viscosity of the fluid had no effect on this simulation result. Similarly, the range of density values tested in this analysis had no effect on the flow velocity within capillaries, nor on the pressure drop from arteriole to venule (data not shown). For the calculation of the static pressure, the conservation of momentum (*Eq. 2*) must be considered. This includes a dependence on viscosity. Correspondingly, the pressure drop showed a linear dependence on both inlet velocity and fluid viscosity (Fig. 5*B*). Although changes in the static pressure at the outlet affected the overall pressure in the model, the pressure drop remained unchanged.

With these dependencies in mind, we compared Newtonian and non-Newtonian viscosity models in the following experiments, keeping the fluid density and boundary conditions constant at their standard values (Supplemental Table S.4).

In summary, we extended our in silico model by including blood flow simulations. We observed a nonmonotonic velocity profile over the ACN. The flow velocity at the inlet has a positive linear effect on both target variables. The viscosity of the fluid has a positive linear effect on the pressure drop. Accordingly, differences in the pressure drop were observed in simulations with Newtonian and non-Newtonian viscosity models.

### Inference of Connection of the ACN to Arterioles from Physiological Measurements

**Figure 6.**
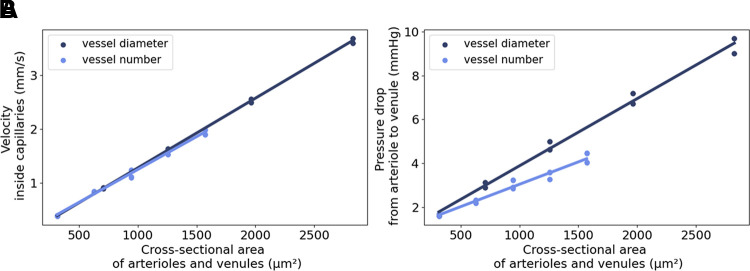
In silico hemodynamic indices from flow simulations in symmetric connectivity models based on the Alveolus_D_ model. The alveolar capillary network model was connected to either a growing number of arterioles and venules, ranging from one to five (light blue), or to one set of vessels with increasing diameters, ranging from 20 µm to 60 µm (dark blue). After computational fluid dynamics simulations of Newtonian fluid flow, the resulting mean velocity at the central position within the alveolar capillary network (ACN) (*A*) and pressure drop from arteriole to venule (*B*) were plotted against the cross-sectional area of the inlet- and outlet vessels. The dots represent the values from the four replicates and the straight lines are the regression curves. [Figure reproduced from a thesis dissertation under CC BY-NC license (69).]

We investigated the linkage between the ACN and the pulmonary vascular tree by comparing models with different configurations of generic arterioles and venules. We performed simulations on four replicates of the Alveolus_D_ sheet-flow model. For each network, we obtained five measurements of the blood flow velocity: at the inlet (set by the boundary condition), in the capillary network close to the transition from arteriole to network, in the center of the network, in the network close to the transition to the venule and at the outlet (Supplemental Fig. S.5*A*). First, symmetrical models were considered in which arterioles and venules had the same configuration. To compare between configurations of vessel number and of diameter (Supplemental Figs. S.5 and S.6), we considered cross-sectional area *A* as a common parameter. Both mean velocity within capillaries and pressure drop from arteriole to venule rose linearly with increasing cross-sectional area of arterioles and venules (Fig. 6). With respect to mean flow velocity, we did not observe differences in this relationship depending on whether the increase in cross-sectional area was due to changes in vessel diameter or the number of vessels. The regression lines, expressed as *v* = −0.01 + 0.00129·*A* and *v* = 0.03 + 0.00122·*A* respectively, closely aligned. Considering the velocity distribution within the ACN, we observed that with increasing cross-sectional area, the velocity variation increases (Supplemental Fig. S.7). Comparing an increase in vessel number with an increase in vessel diameter, we find that the velocity variation increases faster when increasing the vessel number. The velocity ranges for the larger cross-sectional areas were comparable for the two cases. The mean pressure drop exhibited a more pronounced increase with cross-sectional area when the vessel diameter was enlarged (Δ*p* = 0.83 + 0.00306·*A*), as opposed to the increase resulting from a higher number of vessels (Δ*p* = 1.01 + 0.00204·*A*) (Fig. 6*B*).

**Figure 7.**
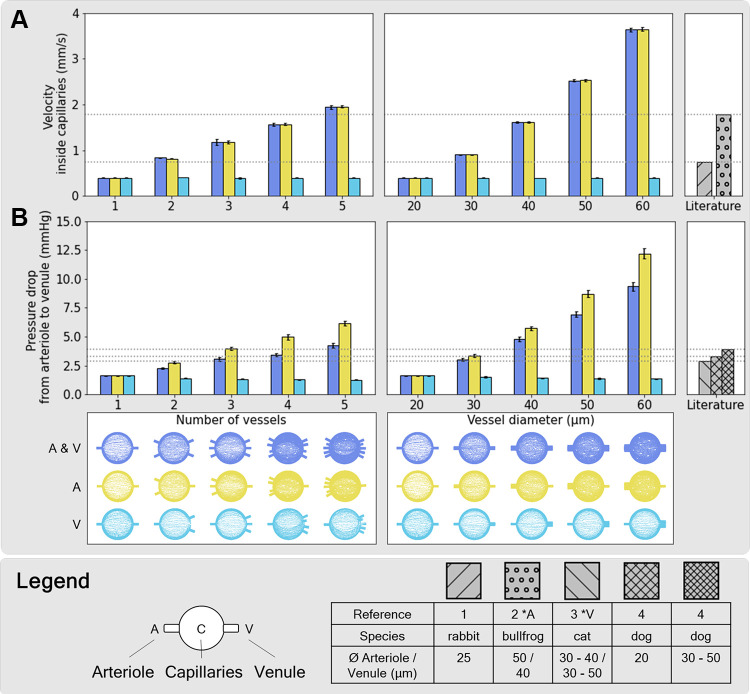
In silico hemodynamic indices from flow simulations in symmetric and asymmetric connectivity models based on the Alveolus_D_ model. Alveolar capillary network models were connected to increasing numbers (*left*) or diameters (*right*) of arterioles (yellow), venules (turquoise), or both (blue). Flow velocity within capillaries (*A*) and pressure drop from arteriole to venule (*B*) were predicted with computational fluid dynamics simulations using a Newtonian viscosity model. The results were compared with literature values of ^1^rabbit (40),^2^bullfrog (38), ^3^cat (39) and ^4^dog (41). *A Simulation boundary condition for inlet velocity at the arterioles were based on a reference from this publication. *V Simulation boundary condition for outlet pressure at the venules was based on references from this publication. We show mean and standard deviation of the values at the centers of the four alveolar capillary network (ACN) replicates. [Figure reproduced from a thesis dissertation under CC BY-NC license (69).]

Next, we included asymmetric models where only one vessel type (arteriole or venule) was configured whereas the other remained in the default configuration. With increasing number or diameter of arterioles, both target values increased (Fig. 7). The exact values are given in Supplemental Tables S.5 and S.6. Regarding the mean velocity within capillaries, we did not observe a difference between symmetrical and asymmetrical arteriolar configuration. The increase in pressure drop, however, was more pronounced in the asymmetric models compared with the symmetric models. Increasing the number or diameter of connected venules alone had no clear effect on the target values. Additional measurements at various positions in the model revealed that the asymmetric configuration of the venules only influenced the flow velocity within the venules, but not within the capillary network (Supplemental Figs. S.5 and S.7).

We compared our results with reference values from different species available in the literature (38–41). These publications include information on the diameters of arterioles and venules (Fig. 7), but the number of these vessels per alveolus is not specified. Our CFD simulation results using the Newtonian viscosity model aligned with the literature values for capillary velocity and arteriole-to-venule pressure drop, despite occasional discrepancies in arteriolar diameters. The comparison suggests that the ACN of a single alveolus is linked to at least two arterioles with diameters of 20 µm or a single arteriole with a minimum diameter of 30 µm. In the results obtained from simulations using a non-Newtonian viscosity model, we made similar observations regarding velocity (Supplemental Fig. S.8*A*). However, the pressure drop was distinctly higher in these simulations (Supplemental Fig. S.8*B*), so that only models with a single 20-µm arteriole provided results in the range of the literature values.

**Figure 8.**
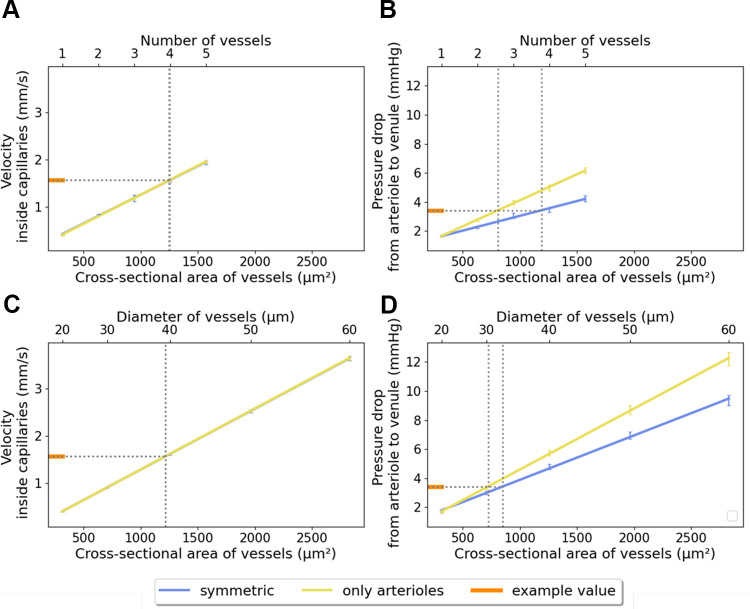
Regression analyses quantify the relationship between target values and the cross-sectional area of arterioles and venules in our models. The example value pair (orange) corresponds to the simulation outcomes from a model with each four arterioles and venules, all with a diameter of 20 µm. The increase in cross-sectional area within the different model set corresponds to changes in either the number (*A* and *B*) or the diameter (*C* and *D*) of only arterioles (yellow) or both arterioles and venules (blue). *A* and *C*: in the case of mean flow velocity within capillaries, the regression lines of the different model sets overlap. *B* and *D*: the regression lines for pressure drop yield identifiable variations. All results are from simulations using the Alveolus_D_ model and the Newtonian viscosity model. The bars indicate mean and standard deviation of the values at the centers of the four alveolar capillary network (ACN) replicates. [Figure reproduced from a thesis dissertation under CC BY-NC license (69).]

Furthermore, we demonstrate how the regression of the simulation results can be used to infer the configuration of arterioles based on measurements of capillary velocity and pressure drop (Fig. 8). Notably, the regression curves for capillary velocity overlap to the extent that distinctions between models with different vessel configurations become indistinguishable. In contrast, the regression curves for pressure drop yield identifiable variations. With these analyses at hand, we aimed to predict the arteriolar cross-sectional area using a pair of example values. In the absence of available experimental data, we selected values from our simulation outcomes, opting for the scenario featuring each four arterioles and venules, all 20 µm in diameter. The corresponding simulation results were a capillary flow velocity of 1.56 mm/s and a pressure drop of 3.44 mmHg. According to the regressions, this velocity value is associated with a cross-sectional area of around 1,200 µm^2^—equivalent to four vessels 20 µm in diameter or single 40-µm diameter vessels (Fig. 8*A*). Regarding the pressure drop value, the distinct regression lines predict different possible cross-sectional area values. The regression analysis of the model set comprising symmetrical variations in the number of vessels points to an area of 1,200 µm^2^. This closely corresponds to a model with four vessels, each 20 µm in diameter and thus aligns with the result obtained from the velocity regression. In addition, this pressure drop value could have been measured in models with an arteriolar cross-sectional area of less than 850 µm^2^, for example in the form of a single 30-µm arteriole and a single 20-µm venule. However, these models can be excluded as they do not agree with the velocity regression analysis. In conclusion, from the mean flow velocity within capillaries we can deduce the vessel cross-sectional area. From the pressure drop regression, we obtain further information on possible configurations of arterioles and venules. In combination, we can identify the model geometry associated with this pair of values. We performed such an analysis with several pairs of values and in most cases obtained two alternative connectivity configurations (e.g., see Supplemental Fig. S.9), differing in whether the arteriolar cross-sectional area is due to the vessel number or diameter. Hence, assuming that experimental measurements for capillary flow velocity and pressure drop from arteriole to venule would be available, we can make an unambiguous or two alternative predictions about the connectivity configuration.

In summary, we found that the target values increase linearly with the arteriolar cross-sectional area. With our current choice of boundary conditions, the simulation results are consistent with the existing literature values for flow velocity and pressure drop. Comparison of the latter with our results from Newtonian simulations suggests that the ACN of a single alveolus is linked to at least two arterioles with diameters of 20 µm or a single arteriole with a minimum diameter of 30 µm. On the other hand, comparing our non-Newtonian simulation results with literature values indicate that the ACN of a single alveolus is connected to no more than one 20-µm arteriole. If corresponding experimental measurements for capillary velocity and pressure drop were available, features of the structure of the connection between the ACN and the vascular tree could be inferred.

### Linking ACN Connectivity to Gas Exchange Efficiency

**Figure 9.**
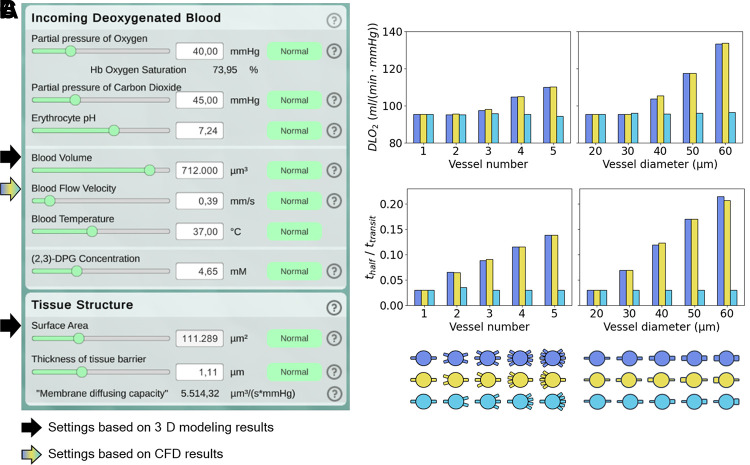
Variations in blood flow impact the results of gas exchange simulations in *Alvin*. *A*: parameter settings in *Alvin* based on the three-dimensional (3-D) morphological modeling (black arrows) and computational fluid dynamics (CFD) simulations (colored arrow). The remaining parameter values were taken from Schmid et al. (17). The blood volume was set to the volume of our sheet-flow Alveolus_D_ alveolar capillary network (ACN) model. The value for surface area was chosen according to the surface area available for gas exchange on our sheet-flow Alveolus_D_ ACN model. The blood flow velocity within capillaries was adjusted based on the CFD results from the different connectivity models (blue, yellow, and turquoise). From the gas exchange simulations in *Alvin*, the following output parameters were analyzed: pulmonary diffusion capacity for oxygen ($D_{{\text{L}}_{{\text{O}}_{2}}}$) (*B*) and reaction half time normalized to transit time (*t*_half_/*t*_transit_) (*C*). Reaction half time is the time point at which 50% of the oxygenation that blood undergoes during its transit along the alveolus is completed. [Figure reproduced from a thesis dissertation under CC BY-NC license (69).]

Gas exchange efficiency is the ultimate function of interest of an alveolus. Therefore, we asked the question: How do the differences in capillary blood flow dynamics observed in our connectivity analyses affect gas exchange efficiency? To address this question, we used our previously develop application *Alvin* that comprises a model for gas exchange at the level of an alveolus (17). The parameter settings in *Alvin* were chosen based on the parameters from the 3-D morphological modeling (Fig. 9*A*, Table 2). The velocity parameter was adjusted to the CFD results for the mean capillary velocity in each case. The simulation results showed that oxygen saturation reached an equilibrium early during blood transit along the capillary. To quantify the efficiency of gas exchange, we analyzed two output parameters in *Alvin*: the pulmonary diffusion capacity for oxygen $D_{{\text{L}}_{{\text{O}}_{2}}}$ (Fig. 9*B*) and the reaction half-time normalized to transit time (*t*_half_ /*t*_transit_, Fig. 9*C*). $D_{{\text{L}}_{{\text{O}}_{2}}}$ quantifies the rate of oxygen consumption V̇o_2_ relative to the average oxygen partial pressure gradient between air and blood ΔPo_2_ (42):

(*4*) $$D_{{\text{L}}_{{\text{O}}_{2}}}=\frac{{\dot{\text{V}}\text{O}}_{2}}{\Delta {\text{PO}}_{2}}.$$

At a blood flow velocity of 0.4 mm/s, which was measured in our default model, we obtained a $D_{{\text{L}}_{{\text{O}}_{2}}}$ of 95.3 mL/(mmHg·min) and a normalized reaction half-time of 0.03 in *Alvin*. As the number or diameter of connected arterioles rises, and with it the blood flow velocity within capillaries, both output parameters also increase. $D_{{\text{L}}_{{\text{O}}_{2}}}$ increases quadratically with the arterial number (*n*) or diameter (*d*) according to $D_{{\text{L}}_{{\text{O}}_{2}}}$ = *a*·(*n*|*d*)^2^ + *b*·(*n*|*d*) + *c* (Supplemental Fig. S.10). For the normalized reaction half-time, this occurs linearly (Supplemental Fig. S.11).

In conclusion, an increase in blood flow velocity in the alveolar capillaries had a positive effect on oxygen diffusion capacity. Although the reaction half-time was reached later as the blood flow increased, it remained within the first quarter of the total transit time.

## DISCUSSION

By synergistically combining data-based 3-D morphological modeling and computational fluid dynamics simulations, we developed an integrative approach to study the morphology and connectivity of the alveolar capillary network.

### Integrative 3-D Modeling and Evaluation of Alveolar Capillary Network Geometry

With regard to the morphological aspect, 3-D in silico modeling supports the concept of the sheet-flow model geometry as an appropriate representation of the ACN. We integrated data from several morphological studies (1, 3, 19–25), using some parameters as a basis for 3-D modeling and the others for model evaluation. The alveolar capillary network was constructed using two concepts, sheet-flow and tube-flow. Both model types yielded volume and surface area values for the capillary network below published estimates. Variations in the data such as these were anticipated. Experimental methods for studying alveolar morphology involve tissue fixation, which inherently introduce bias and loss of information (8). Other uncertainties arise from simplifications like assuming alveoli to be 3/4 spheroids or from having to derive some reference values from other parameters. For example, the volume of the alveolar capillary bed was estimated from the capillary volume of the entire lung (3) and a number of 480 million alveoli in the lung (21). Despite these limitations, the experimental data are extremely valuable and the only source on which we can draw to date. Nevertheless, we argue that the parameters characterizing alveolar morphology should not be considered in isolation. Instead, they should be related to each other, as we have done in our integrative 3-D model. In this way, dependencies among the different parameters (e.g., between diameter or volume of the alveolus and surface area of the ACN) can be quantified and the conclusiveness of the measurements can be assessed.

### CFD Simulation of Blood Flow in Alveolar Capillaries: Assumptions, Model Evaluation, and Viscosity Impact

Incorporating CFD simulations into the sheet-flow geometry allowed us to estimate blood flow paths, velocity, and pressure distribution in three spatial dimensions. Our model is based on several assumptions.

In vivo, breathing dynamics are implemented as a sequential contraction and expansion of the alveoli. We considered a static tissue based on experimental measurements from fixed lungs (Supplemental Table S.1). Approximating breathing dynamics as periodic would result in periodic expansion and contraction of the alveolar capillary network, hence in periodic fluctuations in blood velocity and pressure drop. We expect our static case to approximate the time-average of the dynamic process well. For the alveolar capillary network, we assumed rigid walls since implementation of local deformations are not straight forward. Questions that arise are what kind of deformations occur and are they specific to individual (43).

For the alveolar geometry in our model, we assumed an open 3/4 spheroid with random holes. This is the most common, but not the only alveolar geometry that has been observed (19). Furthermore, diseases like lung fibrosis have been shown to affect the morphology of the ACN such as the size and the number of the pillars and the surface area (44). Our results for different alveolar capillary network replicates indicate that smaller morphological variations such as different random configurations of the whole have a negligible effect on the blood flow dynamics. In the future, it will be interesting to study larger morphological variations due to altered base shapes of the alveolus or due to variations in the ACN thickness and the number and size of pillars.

For blood flow, we assumed a continuous fluid in alignment with previous approaches (45, 46). In tubes, this continuum assumption primarily affects the velocity profile (47). For a continuous Newtonian fluid with no-slip boundary conditions, the velocity decrease from the center of a tube to the walls is steeper than if individual red blood cells are considered in the fluid. For a capillary with a diameter of 20 µm, the maximum velocity at the center of the continuum assumption leads to an overestimation of 20% (47). For mean velocities as considered in our model, the discrepancy is much less. In addition, the alveolar capillary network, which constitutes the majority of our system, was modeled as a sheet. The differences for this geometry are not yet known, but we expect them to be similar or smaller than those for the tube. Hence, the continuum fluid assumption may slightly overestimate mean velocities, potentially causing a slight underestimation of the cross-sectional area. In the model evaluation, we observed a linear correlation between the inlet velocity boundary condition and our target values—mean flow velocity within capillaries and pressure drop from arteriole to venule. In addition, the pressure drop was dependent on the fluid viscosity. Consequently, comparison between simulations with Newtonian and non-Newtonian viscosity models yielded comparable velocity values (around 0.4 mm/s) but considerable differences in pressure drop (1.64 mmHg with a Newtonian fluid vs. 3.54 mmHg with a non-Newtonian fluid). In general, the results showed that the flow direction parallel to the inlet and outlet vessels was strongly preferred. Our simulation results are consistent with published in vitro measurements. Stauber et al. (48) have developed a device that mimics the sheet-flow ACN. For 7-µm interpillar distance, the closest match to our sheet-flow model, they have measured a red blood cell (RBC) flow velocity of 0.3 mm/s at a pressure gradient of 0.5 kPa (3.75 mmHg). Previous theoretical work has predicted distribution of blood flow in line with our results (46). Their 3-D hollow sphere model compares well with our sheet-flow ACN models, except that the geometry is based on mouse morphometrics and is more simplified. They observed a decrease in flow velocity in the middle of the capillary bed and higher velocities near the inlet and outlet surfaces, and a continuous pressure drop from inlet to outlet. Burrowes et al. (16) have predicted blood flow in 3-D tube-flow ACN models across several alveoli, using a fixed pressure drop of 8 cmH_2_O (5.88 mmHg) as a boundary condition. The mean flow velocity, derived from transit times, ranged from 0.15 mm/s to 1.07 mm/s for different transmural pressure conditions. In summary, the flow velocity within capillaries in our simulations lay within the range of references from previous theoretical and in vitro studies. Furthermore, the reference values for arteriole-to-venule pressure drop were undercut in our CFD simulations with constant viscosity but closely approximated in simulations with the non-Newtonian viscosity model. The selection of a 2 cP constant viscosity in our Newtonian CFD simulations was based on estimated apparent viscosities of blood in pulmonary capillaries from several studies (49–52). In selecting the non-Newtonian viscosity model and the corresponding parameters, we followed the approach of Albors et al. (37) who used this model to simulate blood flow in the left atrium. However, it covers a range of viscosity values between 3.5 cP and 56 cP which is above the estimated apparent viscosity in the pulmonary capillaries. In the future, it is advisable to use a viscosity model that accurately represents blood’s rheological properties within the microvasculature, where factors like hematocrit and vessel diameter exert a strong influence.

To determine the most suitable model assumptions, including but not limited to the boundary conditions and the viscosity model, we rely on a consistent dataset, ideally derived from the same experiment or, at a minimum, from the same species. Currently, literature values for the choice of boundary conditions and as benchmarks for comparison with the simulation results have to be taken from several studies working with different species. These physiological metrics sometimes show large differences between different species. This is complicated by the fact that these measurements were performed in vessels of varying diameters. The optimal data set would include blood flow velocities in arterioles and capillaries, blood pressure in arterioles and venules, and the mean diameters of the vessels in which these values were measured.

In summary, we developed a base model for simulating blood flow in the pulmonary capillaries. Qualitatively, our simulation results are consistent with those from previously published in vitro studies and blood flow simulations in the literature. However, we had to make a number of assumptions. Furthermore, we observed strong dependencies of our target values on the inlet velocity boundary condition and/or the fluid viscosity. To refine and validate our model, further computational studies in combination with appropriate experimental data are required.

### Investigating ACN Connectivity with the Vascular Tree: A Novel Approach Linking Morphology and Function

For connectivity analyses, we used our integrative in silico approach to investigate the connection of the ACN to arterioles and venules. The CFD results for mean flow velocity inside the capillary bed and the pressure drop from arteriole to venule in our connectivity models were within the range of published reference values.

Comparison of our simulation results on flow velocity with literature references suggests that a single alveolar ACN is likely connected to at least two arterioles with diameters of 20 µm or a single arteriole with a diameter of at least 30 µm. The pressure drop results depended on the viscosity model. With constant low viscosity (Newtonian model), a similar prediction can be made about the arteriole configuration as for velocity simulation results. In contrast, simulations with the non-Newtonian model indicate that the ACN around an alveolus is connected to no more than one 20-µm arteriole.

Previous studies that have examined the linkage between arterioles, venules, and the ACN in the human lung have done so from a morphological perspective (29, 31, 32). Across these studies, naming of the smallest pulmonary vessels is inconsistent, but considering vessel diameter allows for direct comparison. These findings suggest that one arteriole with a diameter of ∼20 µm connects to multiple alveoli, with values ranging from 4.8 (32) to 24 (29). For the actual connection between the vascular tree and the capillary network, so-called precapillary vessels have been introduced (29, 31). Only vague information is provided about these vessels, such as that their diameters approach the capillary diameter.

The morphological estimates suggest that in our simulations we should observe an oversupply of the capillary network around a single alveolus already by a single 20-µm arteriole. However, our results do not indicate this. Once experimental data become available to further verify our CFD models, it will be intriguing to examine whether the predictions from morphological studies still hold from a functional point of view.

Our current model shows possibilities for predicting morphological features based on physiological parameters. If consistent experimental measurements for flow velocity and pressure drop were available, details of the arteriole and venule configuration could be inferred. Depending on the values, we can identify two possible connectivity models based on either the number or the diameter of arterioles or we can make a unique prediction.

Previous theoretical studies on pulmonary blood flow focused on predicting physiological parameters based on morphological properties. The work of Zurita and Hurtado (46) has predicted the 3-D spatial distribution of blood flow and gas exchange. Multiscale models (53–56) are more complex and allow to investigate even broader relationships. They enable predictions of both whole lung function with alterations in small vascular structures and small-scale vascular function in response to impairment of the proximal pulmonary artery. Theoretical studies with CFD blood flow simulations for other organs such as the heart are even more elaborated (57). Mill et al. (58, 59) and Pons et al. (60) have used CFD simulations with patient-specific boundary conditions and dynamic mesh motion of the 3-D geometry to assess thrombus risk in the left atrial appendage, showcasing the clinical potential of CFD in hemodynamic studies.

The novelty in our approach is that we exploit the interdependence of structure and function in the opposite direction: On the basis of physiological blood flow parameters, we evaluated the realism of systematically introduced morphological variations. To our knowledge, such an inverse approach on structure-function relationships has only ever been used on a cellular level, inferring position and morphology of neurons from in vivo extracellular voltage recordings using biophysical modeling (61, 62).

### Extension to Gas Exchange

To link the outcomes of our 3-D modeling and blood flow simulations to gas exchange efficiency, we used our interactive model *Alvin* (17) and predicted the oxygen diffusion capacity $D_{{\text{L}}_{{\text{O}}_{2}}}$ for the different connectivity scenarios. For our default connectivity model, we calculated a $D_{{\text{L}}_{{\text{O}}_{2}}}$ of 95.3 mL/(mmHg·min). This value falls right in the middle between the physiological estimate of 30 mL/(mmHg·min) (63) and the morphological estimate of 158 mL/(mmHg·min) (64).

Under normal conditions, the partial pressures of respiratory gases reach equilibrium early during blood transit through the alveolar zone (65). Our results align with this observation, showing that even at higher blood flow velocities, the reaction half-time remains within the first quarter of the transit time, highlighting blood flow as the limiting factor for oxygen uptake. If the reaction were diffusion-limited, the respiratory gases would not reach equilibrium by the end of transit.

In summary, our model makes predictions about the course of gas exchange under normal conditions. Previous computational approaches have directly coupled calculations of ventilation and gas exchange (66), of perfusion and gas exchange (46) or even of ventilation, gas exchange, and perfusion (45, 67, 68) in their simulations. So far, we integrated intermediate results in *Alvin*. In future studies, we are planning to expand this approach by a more detailed model for gas exchange. At this stage, our approach already benefits from the morphological detail and the accessibility of the gas exchange simulation gained through interactivity.

### Conclusions and Outlook

In conclusion, our integrative in silico modeling approach complements previous studies and offers a unique perspective on the structure-function relationship of the pulmonary microvasculature. By synergistically combining data-based 3-D modeling and computational fluid dynamics simulations, we successfully refined our understanding of the alveolar capillary network and its relationship with the vascular tree. Moving forward with appropriate experimental data for validation and incorporating a suitable viscosity model to account for blood’s rheological behavior, our in silico model holds promise for more precise simulations and further insights into microvascular hemodynamics. In addition, incorporation of the modeling results into our interactive application for gas exchange analyses (17) demonstrates the potential of our approach to link morphological features to gas exchange efficiency. For future research, introducing dynamic changes in alveolar volume over time will add complexity to the model, enhancing its fidelity to real physiological scenarios. The same holds true for the combination of several alveoli and their mechanical and hemodynamic interdependence. Furthermore, automating the morphological modeling process would facilitate more efficient exploration of systematic geometric variation.

On a final note, our approach demonstrates the feasibility of deriving morphological aspects from physiological parameters in the case of blood flow simulations in the pulmonary microvasculature, a new strategy that is transferable to other tissues and organs.

## DATA AVAILABILITY

Meshes used in this study are openly available at https://doi.org/10.5281/zenodo.13320075. Please note that due to size restrictions, we uploaded four exemplary files. All other meshes are available from sabine.fischer@uni-wuerzburg.de.

## SUPPLEMENTAL MATERIAL

10.5281/zenodo.13320075 Supplemental Figs. S.1–S.11, Supplemental Tables S1–S6, and Supplemental Sections S.1–S.5: https://doi.org/10.5281/zenodo.13320075.

## GRANTS

K.S. was supported by an EMBO Scientific Exchange Grant 9271. K.S. and S.C.F. acknowledge the support by Universitätsbund Würzburg Grant AZ21-16. A.C.H. was supported by German Research Foundation Grant DFG, SFB-TR 84, B6, Z1a as well as by the Einstein Foundation (EC3R) Charité 3R. W.M.K. and M.O. were supported by the German Research Foundation Grant DFG, SFB1449, B01.

## DISCLOSURES

Wolfgang Kuebler is an editor of *American Journal of Physiology-Lung Cellular and Molecular Physiology* and was not involved and did not have access to information regarding the peer-review process or final disposition of this article. An alternate editor oversaw the peer-review and decision-making process for this article. None of the other authors has any conflicts of interest, financial or otherwise, to disclose.

## AUTHOR CONTRIBUTIONS

K.S., A.L.O., A.C.H., and S.C.F. conceived and designed research; K.S. performed experiments; K.S. analyzed data; K.S., A.L.O., O.C., W.M.K., M.O., A.C.H., and S.C.F. interpreted results of experiments; K.S. prepared figures; K.S. drafted manuscript; K.S., A.L.O., O.C., W.M.K., M.O., A.C.H., and S.C.F. edited and revised manuscript; K.S., A.L.O., O.C., W.M.K., M.O., A.C.H., and S.C.F. approved final version of manuscript.
